# Oxidative Stress as a Link between Cerebrocardiovascular and Psychiatric Disorders

**DOI:** 10.1155/2020/5685317

**Published:** 2020-02-07

**Authors:** Maria Luca, Tomasz Guzik, Antonina Luca

**Affiliations:** ^1^Department of Medical, Surgical Sciences and Advanced Technologies “GF Ingrassia”, University of Catania, Italy; ^2^Department of Internal and Agricultural Medicine, University Medical College, Cracow, Poland; ^3^Institute of Cardiovascular and Medical Sciences, University of Glasgow, UK

Neuropsychiatric disturbances show high rates of comorbidity with cerebrocardiovascular disorders. In particular, depression and anxiety independently predict both cerebrovascular and cardiac events, which in turn represent a major cause of mortality in individuals suffering from psychiatric illnesses. On the other hand, depressive symptoms are extremely common in poststroke and vascular dementia.

From a molecular point of view, oxidative stress (OS) could not only represent the major contributor for the pathogenesis and progression of psychiatric and vascular disorders but also explain the high rates of comorbidity between these disorders.

This special issue is aimed at improving the current knowledge about the role of OS in the occurrence and progression of neuropsychiatric and cerebrocardiovascular disorders.

Three papers focus on the role of OS in diabetes-related complications. In particular, P. Yang et al. reviewed current evidence on the role of the advanced glycosylation end products in the occurrence of OS-related cardiovascular complications in diabetes. N. Palachai et al. demonstrated the beneficial role of mulberry and ginger in reducing the metabolic alterations as well as the OS status and proinflammatory cytokines in male Wistar rats with metabolic syndrome. Finally, J. Wattanathorn et al. demonstrated the antioxidant and anti-inflammatory effect of *Oryza sativa* and *Anethum graveolens* in male Wistar rats with metabolic syndrome.

Two articles explore the detrimental effect of stress on health. More specifically, O. Hahad et al. reviewed the damaging effect of environmental noise on mental and cerebrocardiovascular health in relation to OS, with special focus on the autonomic nervous system, endocrine signaling, and vascular dysfunction. Instead, D. C. Wigger et al. studied the link between early life stress and the risk of cardiovascular disorders evaluating the expression of the myocardial oxytocin receptor and the enzyme cystathionine *γ*-lyase.

Two reviews summarize current knowledge on the relationship between cerebrocardiovascular disorders and neuropsychiatric disturbances. In particular, D. Lin et al. described the common OS pathways and risk factors shared by ischemic cardiocerebrovascular disorders and depression. M. Luca and A. Luca focused on the role of OS-related endothelial damage in the pathogenesis of both vascular depression and cognitive impairment, also commenting on the beneficial effect of aerobic physical exercise on these disorders.

Several authors studied the role of OS from a multidisciplinary point of view. In detail, L. Venturini et al. performed a pilot study supporting the anti-inflammatory and antioxidant effects of probiotic administration on chronic fatigue syndrome/myalgic encephalomyelitis. M. Castaldo et al. demonstrated that SH-SY5Y cells, whose proliferation, migration, and neurite outgrowth are improved by formyl peptide receptor-1, can stimulate NOX-dependent superoxide generation. N. Yu et al. reported the therapeutic effect of *Ganoderma lucidum triterpenoids*, an inhibitor of the ROCK signaling pathway, in improving cognitive performance and reducing hippocampal cell apoptosis in Alzheimer's disease model mice.

We believe that these contributions provide an updated and comprehensive view on the role of OS in neuropsychiatric and cerebrocardiovascular disorders.

